# Chronic voice disorder after coronavirus disease 2019 infection and its treatment using the cricothyroid visor maneuver: a case report

**DOI:** 10.1186/s13256-023-03780-w

**Published:** 2023-02-26

**Authors:** Narges Moein, Ali Dehqan, Ronald C. Scherer

**Affiliations:** 1 Department of Speech Language Pathology, School of Rehabilitation Sciences, Iran University of Medical Sciences, Madadkaran St., Shahnazari Ave., Mirdamad Blvd., Madar Sq., Tehran, Iran; 2 Rehabilitation Sciences Research Center, Zahedan University of Medical Sciences, Zahedan, Iran; 3 Department of Communication Disorders, Bowling Green State University, Bowling Green, OH USA

**Keywords:** COVID-19, CVM, Manual therapy, Dysphonia, Voice therapy

## Abstract

**Background:**

Regarding human coronavirus, the severe acute respiratory syndrome coronavirus 2 pandemic, the novelty of disease, and consequently the lack of studies, the etiology of dysphonia in patients with coronavirus disease 2019 is still unknown and needs to be investigated. The purpose of the current study is to investigate the effect of a new manual therapy technique, cricothyroid visor maneuver, on muscle tension dysphonia symptoms for a patient who had experienced dysphonia symptoms due to the coronavirus disease 2019 infection.

**Case presentation:**

A 55-year-old retired Iranian teacher who was diagnosed with muscle tension dysphonia by an otolaryngologist participated in this study. Fifty days before being referred to an otolaryngologist, he was diagnosed with coronavirus disease 2019 on the basis of the results of a standard laboratory test, namely real-time polymerase chain reaction. Treatment was provided in ten sessions. Pre- and post-treatment audio recordings of sustained vowels, selected sentences, and connected speech samples were submitted for auditory perceptual and acoustic analysis to assess the effects of the treatment program. Also, videolaryngostroboscopy voice quality perceptions by the patient, both before and after therapy, were assessed. The reduction in all features of the Consensus Auditory-Perceptual Evaluation of Voice was observed. The results of acoustic assessment showed that jitter (35.13%) and shimmer (20.48%) decreased; moreover, the harmonics-to-noise ratio (1.17%), cepstral peak prominence smoothed (28.53%) and maximum phonation time (15.5%) increased after treatment sessions. The scores of four parameters of Stroboscopy Examination Rating Form (SERF) form changed after cricothyroid visor maneuver therapy. Also, the visual analog scales score at the pre-treatment assessment was 40, and increased to 90 at the post-treatment assessment.

**Conclusions:**

The effectiveness of cricothyroid visor maneuver therapy on dysphonia associated with coronavirus disease 2019 was investigated in the current study. This case study has highlighted chronic dysphonia after coronavirus disease 2019 infection, and suggests that the cricothyroid visor maneuver therapy approach may have positive outcomes for patients with muscle tension dysphonia with this background.

## Introduction

Human coronavirus, severe acute respiratory syndrome coronavirus 2 (SARS-CoV-2), is a highly pathogenic virus that was first identified in late 2019 and causes coronavirus disease 2019 (COVID-19) [1]. The World Health Organization (WHO) declared a pandemic in March 2020. The SARS-CoV-2 virus affects the respiratory system, and its most common symptoms are fever, coughing, headaches, pneumonia, and loss of taste and smell [2]. In patients with severe COVID-19, the respiratory system is highly affected, requiring intubation and tracheostomy [3]. Prolonged intubation can result in significant laryngeal complications, such as abnormalities relative to the mucosal wave, periodicity, glottal closure, symmetry of motion of the two vocal folds, unilateral vocal fold immobility, and posterior glottic and subglottic stenosis. These complications can lead to voice and swallowing difficulties [4]. On the other hand, in less severe cases of COVID-19 where intubation is not used, some breathing problems, coughing, and throat clearing can be observed. These symptoms cause vocal fold damage, such as edema or inflammation [5]. In the study of Lechien *et al*., the prevalence of dysphonia in patients with mild-to-moderate COVID-19 was 26.8%. They also found a significant positive association between dysphonia and coughing [5].

Because of the novelty of the COVID-19 disease and the lack of studies, the etiology of dysphonia in patients with COVID-19 is still unknown and needs to be investigated [5]. One of the effective methods to investigate the etiology of dysphonia is videolaryngostroboscopic examination [6]. Regarding the results of videolaryngostroboscopic examination, four groups of patients can be diagnosed: patients with benign vocal fold lesions, malignant vocal fold lesions, neuromuscular/skeletal disorders of the larynx, or functional disorders of the larynx [6].

Muscle tension dysphonia (MTD) can act as a bridge between functional and organic disorders, and is caused by excessive tension of the (para)laryngeal muscles [7]. Two types of MTD include primary MTD, in which a dysphonia exists in the absence of organic vocal fold pathology, and secondary MTD, in which there is an underlying organic condition [8]. Upper respiratory tract infections and excessive coughing or throat clearing can result in primary MTD [9].

The effective treatment for MTD is voice therapy [10, 11]. The voice therapy for MTD includes direct and indirect aspects. Indirect treatment techniques focus on psychological aspects, such as education about vocal function, vocal hygiene programs, and auditory training. Direct treatment techniques focus on mechanical and physical aspects, such as chewing, yawn-sigh, and laryngeal manipulation [12, 13]. One of the laryngeal manipulation methods is the cricothyroid visor maneuver (CVM) method introduced in recent years [14, 15]. The CVM is used for increasing cricothyroid activity by phonation of high-pitch sound along with cricothyroid visor manipulation. Manipulation of the cricothyroid joint results in the reduction of the duration of therapy, compared with similar therapies [16]. As there are some persistent respiratory complications following COVID-19 infection, and optimal treatment still remains unclear [17–19], it is hypothesized that the dysphonia stemming from COVID-19 may be more resistant to therapy due to the tendency for COVID-19 symptoms to last an unusually long time. The purpose of the present study was to investigate the effect of CVM on MTD symptoms for a patient who had experienced symptoms due to COVID-19 infection.

## Case presentation

The participant was a 55-year-old retired Iranian teacher who was diagnosed with MTD by an otolaryngologist. Fifty days before referring to an otolaryngologist, he was diagnosed with COVID-19 on the basis of a standard laboratory test reverse transcription real-time polymerase chain reaction (rt-PCR) [17–19]. The patient did not have any history of voice disorders, or diagnosis of MTD, before he was diagnosed with COVID-19. He was not a smoker or an alcohol drinker. Additionally, there was no family history of voice problems, or any kind of dysphonia. According to the fact that he was retired and spent a lot of his time at home with his small family, he did not use his voice excessively.

His COVID-19 symptoms were fever, continuous dry cough, headache, and tiredness. Ten days after the onset of disease (appearance of symptoms), all symptoms of his moderate COVID-19 disease were recovered without hospitalization and medications, and his rt-PCR test was negative. The severity of the participant’s COVID-19 was moderate, and he was not hospitalized. Therefore, no information such as a pulse, blood pressure, laboratory findings, and so on had been recorded. At that time (the early stage of the COVID-19 pandemic), because of the large number of patients with severe disease who needed to be hospitalized and because of the shortage of medications, the physicians did not give medications to patients with moderate disease. Thus, the subject of the present study did not take any medications. Nevertheless, he experienced moderate hoarseness for 50 days, and finally went to an otolaryngology clinic.

### Clinical findings

According to the results of the assessment and videolaryngostroboscopy, the otolaryngologist diagnosed MTD and referred the patient to a speech-language pathologist (principal investigator).

### Timeline

A case study was conducted to investigate the effect of the CVM technique on chronic MTD after COVID-19 infection. Auditory perceptual, acoustic, and endoscopic imaging assessments were performed before the treatment sessions, to provide baseline measurements. Then, the participant received ten CVM therapy sessions on alternating days (3 days a week). Immediately after the last session of treatment, the post-treatment assessments were carried out in the same way as the baseline assessments.

### Diagnostic assessment

In this study, four common approaches for clinically assessing the various aspects of voice production were used to perform the pre- and post-treatment assessments. These assessment modalities included (1) auditory perceptual assessment, (2) acoustic assessment, (3) endoscopic imaging of the vocal folds, and (4) voice self-assessment [20].

### Auditory perceptual assessment

To perform an auditory perceptual assessment, the Consensus Auditory-Perceptual Evaluation of Voice (CAPE-V) was used. The CAPE-V is a subjective tool for clinical assessment of voice quality. In this tool, six voice-quality features are evaluated, namely overall severity, roughness, breathiness, strain, pitch, and loudness. The subject performed three tasks consisting of (i) sustained vowels, (ii) reading sentences, and (iii) spontaneous conversation. His voice was assessed using a 100 mm line scale, according to each of the six features. In this visual scale, a score of 0 reflects a voice with normal quality, and a score of 100 represents a voice with severely deviant quality [21]. In 2014, Salary Majd *et al*. investigated the validity and rater reliability of the Persian version of the CAPE-V. The result showed that intraclass correlation coefficient values ranged from 0.32 to 0.85 for inter-rater reliability, and from 0.42 to 0.86 for intra-rater reliability. Regarding the results, it was suggested that the Persian version of the CAPE-V is a valid and reliable scale for auditory perceptual assessment of voice quality [22]. At pre- and post-treatment timepoints, the participant recorded the vowels /a/and /i/ three times each, read six specific sentences one time, and produced 2 minutes of spontaneous speaking. Listeners can judge a patient’s voice by listening to sustained vowels, in which there are no articulatory influences. The second task consists of different sentences with different phonetic contexts, which provide an opportunity to assess different aspects of vocal quality such as coarticulatory influence of vowels, soft glottal attacks, voiced stoppages/spasms, and hyponasality [21]. Then, two speech-language pathologists, each with at least 5 years of experience performing voice therapy and considered as independent and blinded raters, rated all voice samples. The voice samples were presented in such a way that the vowels were first, then the sentences, and then the spontaneous speech.

### Acoustic assessment

Because of the inherent nonstationarity of the continuous speech signal, it is a challenge to acoustically analyze continuous speech [23]. Thus, the sustained vowels were used for the acoustic assessment. In the pre- and post-treatment assessments, the participant was asked to produce a sustained /â/ three consecutive times (with an inhalation between productions), for at least 3 seconds, at a constant and comfortable pitch and loudness. The participant’s voice was recorded in a silent room, through a laptop computer (LENOVO IdeaPad 520-15IKB Type 81BF, manufactured for Lenovo PC HK Limited, China) and a microphone (Sony-220FV) with a frequency band of 100–12,000 Hz that was positioned approximately 10 cm from his mouth. The ambient noise level was measured with a sound level meter (model: TECPEL, DSL-331, New Taipei City, Taiwan), with room noise measured as minimum LA (L is abbreviation of Level. Using “A” is for all acoustic measurements under 100 dB) 27.89 dB and minimum LC (L is abbreviation of Level. Using “C” is for all acoustic measurements are above 100 dB) 38.78 dB. The recording and acoustic analysis of the participant’s productions were performed using Praat software [24]. The middle 1000 milliseconds of each sustained vowel were used for analyses. The three token values were averaged to get the reported value for each measure. Four acoustic measures were used to objectively assess voice perturbations, including jitter (%), shimmer (%), harmonics-to-noise ratio (HNR) (dB), and cepstral peak prominence smoothed (CPPS) [20]. CPPS is a sensitive measure of vocal quality disturbance, and correlates with perceptual judgments of dysphonia severity [25].

In a previous review study, three aerodynamic-related measures, maximum phonation time (MPT), vital capacity (VC), and phonation quotient (PQ) were surveyed. The result showed that MPT was the most sensitive measure among the three for evaluating and monitoring voice changes [26]. Thus, in this study, MPT was also investigated in the pre- and post-treatment assessments.

### Endoscopic imaging

Videolaryngostroboscopy examination can provide imaging information such as mucosal wave, the symmetry of vibration, glottal closure, glottal area variation, and so on. In this study, a videolaryngostroboscopy assessment was conducted using a FIEGERT LED Stroboscopy system. During the imaging, the patient was asked to produce a sustained /i/ at a comfortable pitch and loudness. This assessment was performed at the pre- and post-treatment time points. Two experienced speech-language pathologists who specialize in voice, with at least 5 years of stroboscopy rating experience, used the Stroboscopy Examination Rating Form (SERF) form [27] to rate the stroboscopic imaging. They rated independently and did not know which timepoint each image belonged to.

### Voice self-assessment

Visual analog scales (VAS) are practical, valid, and reliable scales to measure the changes of moods and sensations when the word cannot describe the subjective experience exactly [28, 29]. VAS include a 10 cm line on either end of which the words describe the maximal and minimal extremes of the variable being measured. Subjects are asked to mark a point on the line where it appropriately describes their feelings. Then, the distance from the minimal endpoint to that point is measured using a millimeter tape measure [28]. In this study, the VAS was used as a voice self-assessment tool to compare the voice quality from the patient’s point of view at the pre- and post-treatment timepoints. Thus, the patient was asked to mark a point on the line where it appropriately describes his feelings about his voice quality, from best quality at the right end to worst quality at the left end.

### Therapeutic intervention

In this study, the manual therapy program cricothyroid visor maneuver (CVM) [14, 15] was used with the intent to reduce the excessive tension of the laryngeal muscles and improve voice production. The treatment was performed for ten 30 minute sessions on alternating days (3 days per week). This technique has seven steps that should be performed in the recommended sequence. The steps are presented in Table 1 [14, 15].

**Table 1:** The cricothyroid visor maneuver technique steps

Steps	Step description
1.	The hyoid bone is encircled with the thumb and index finger, which is worked posteriorly until the tips of the major horns are felt
2.	Light pressure is exerted with the fingers in a circular motion over the horns of the hyoid bone
3.	The procedure (1 and 2) is repeated, beginning from the thyroid notch and working posteriorly
4.	The posterior borders of the thyroid cartilage, just medial to the sternocleidomastoid muscles, are located, and the procedure is repeated
5.	With the fingers over the superior borders of the thyroid cartilage, the larynx is worked downward, and moved laterally at times
6.	With the thumbs of both hands over both sides of the visor, the structure is pulled away from both sides for opening of the visor
7.	The patient is asked to produce the vowel /a/ in a high-pitched voice for activating the cricothyroid muscle, as the larynx is worked downward. The patient is also asked to hum or prolong vowels, and any changes in vocal quality are noted. The improved voice was progressively shaped from vowels and words (i.e., counting, stating the days of the week), to short phrases, to sentences, and finally to conversation

### Follow-up and outcomes

All ten 30 minute sessions were without incident, with full compliance of the client.

### Results of auditory perceptual assessment

The overall severity of CAPE-V was calculated at the pre- and post-treatment timepoints independently by two raters. At the pre-treatment timepoint, both raters indicated that the patient’s voice was moderately deviant from normal, and both indicated at the post-treatment timepoint that the quality of the patient’s voice was mildly deviant. The reduction in the degree of deviance was observed in all features of the CAPE-V assessment form. The results are presented in Table 2.

**Table 2:** The consensus auditory-perceptual evaluation of voice pre- and post-treatment scores by two blinded raters

	Rater 1		Rater 2	
Features	Pre-treatment	Post-treatment	Pre-treatment	Post-treatment
Vocal roughness	40	20	42	23
Breathiness	20	9	20	10
Strain	47	22	45	25
Pitch	35	10	30	8
Loudness	20	8	20	10
Overall (severity)	45 (moderate)	15 (mild)	48 (moderate)	17 (mild)

Consensus auditory-perceptual evaluation of voice scores range from 0 to 100, with higher scores indicating more severe dysphonia

### Results of acoustic assessment

The voice of the patient was acoustically assessed at the pre- and post-treatment timepoints. The results showed that jitter and shimmer decreased after the treatment sessions, which suggests an improvement in voicing “instability” and voice quality. Also, the CPPS increased after treatment, also suggesting improved voice quality than before treatment. The HNR remained essentially unchanged between the pre- and post-treatment timepoints. The acoustic data are presented in Table 3. The MPT was assessed at the pre- and post-treatment timepoints. The results showed that the MPT increased (improved) from 13.6 seconds at the pre-treatment assessment to 15.7 seconds at the post-treatment assessment.

**Table 3:** Jitter, shimmer, harmonics-to-noise ratio, and cepstral peak prominence smoothed at the pre- and post-treatment timepoints

Acoustic parameters	Pre-treatment	Post-treatment	Change (%)
F0 (Hz)	112.81	114.22	1.24
Jitter (%)	1.85	1.2	−35.13
Shimmer (%)	6.15	4.89	−20.48
HNR (dB)	12.61	12.76	1.17
MPT (seconds)	13.61	15.72	15.5
CPPS (dB)	11.53	14.82	28.53

F0 (Hz): Fundamental Frequency; HNR (dB): Harmonic to Noise Ratio; MPT (Seconds): Maximum Phonation Time; CPPS (dB): cepstral peak prominence

### Results of endoscopic imaging

**Fig. 1:**
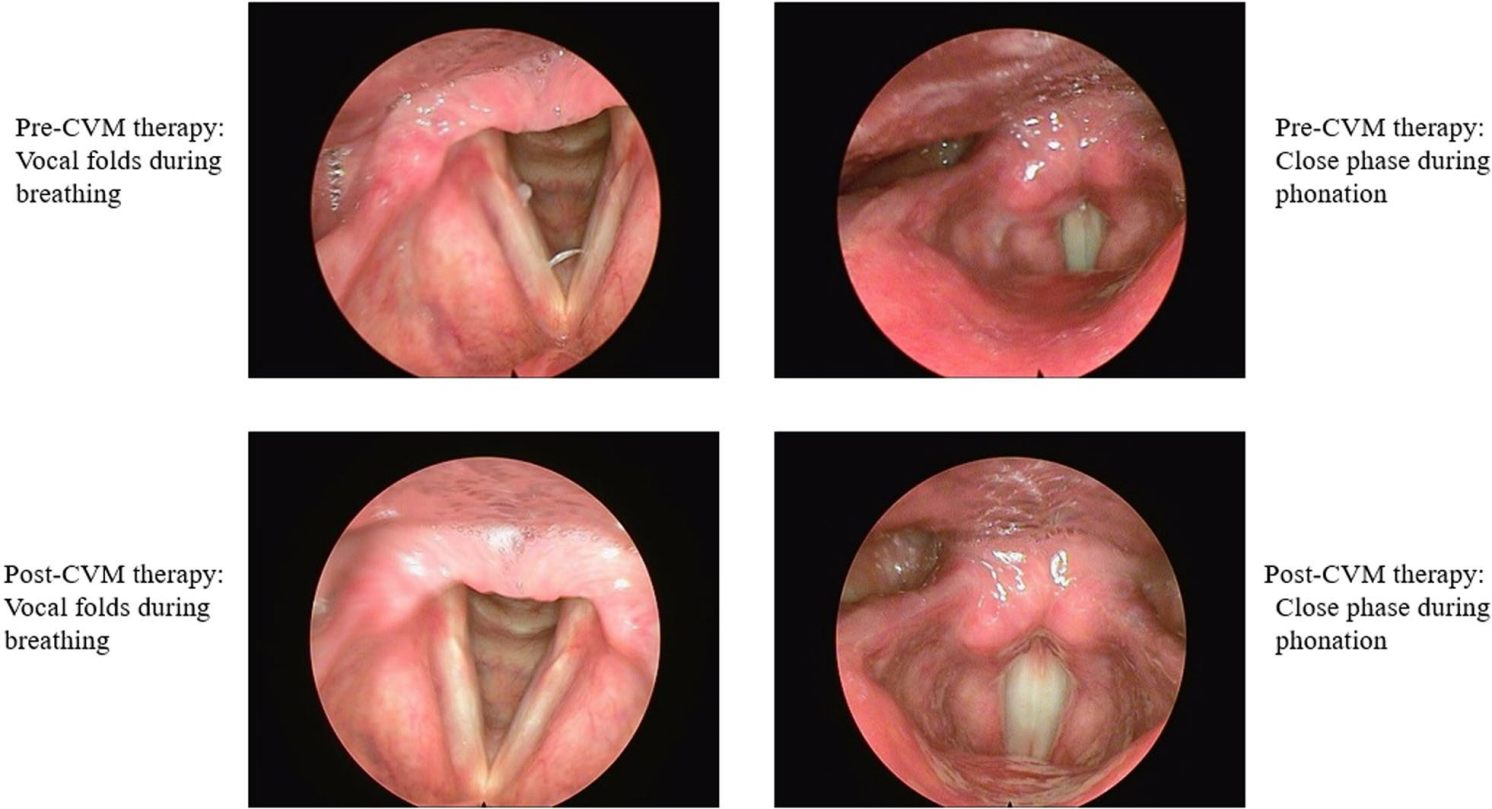
The pictures of vocal folds at the pre- and post-cricothyroid visor maneuver therapy assessments: mediolateral and anterior–posterior compression have been reduced after cricothyroid visor maneuver therapy

According to the SERF form filled out independently by the two judges, the scores of four parameters changed after CVM therapy, including amplitude of vocal fold vibration, mucosal wave, non-vibration portion, and supraglottic activity. These scores showed that, at the pre-treatment assessment, there was mediolateral (ML) and anterior–posterior (AP) compression, which indicated MTD 3 [30]. After the CVM treatment, the scores of the SERF form indicated a decrease in ML and AP contraction as well as an increase in the mucosal wave and vocal fold amplitude of vibration. These results can be observed in Table 4. Also, the pre- and post-treatment stroboscopic images are shown in Fig. 1.

**Table 4:** Stroboscopy parameter for two blinded judges at the pre- and post-treatment assessments

SERF parameter	Pre-treatment		Post-treatment	
	Judge 1 (%)	Judge 2 (%)	Judge 1 (%)	Judge 2 (%)
Glottal closure	100	100	100	100
Amplitude				
Right	40	45	80	80
Left	40	45	80	80
Mucosal wave				
Right	35	40	55	60
Left	35	40	55	60
Non-vibration portion				
Right	20	25	10	10
Left	20	25	10	10
Supraglottic activity				
AP	75	75	25	20
ML	70	75	20	20
Edge smoothness				
Right	0	0	0	0
Left	0	0	0	0
Edge straightness				
Right	0	0	0	0
Left	0	0	0	0
Vertical level	0	0	0	0
Phase closure	66	66	66	66
Phase symmetry	100	100	100	100
Regularity	100	100	100	100

*AP* anterior–posterior, *ML* mediolateral

### Results of voice self-assessment

The VAS self-assessment score by the patient at the pre-treatment assessment was 40, and it was 90 at the post-treatment assessment. The increase of the VAS score suggests that the voice quality improved from the patient’s point of view. The results are limited to immediate effects after therapeutic sessions, and the effects were not followed for 6 months after the treatment.

## Discussion

This case study was conducted to explore the effectiveness of the CVM technique for a patient with MTD after a COVID-19 infection. The impact of this technique was investigated by auditory perceptual assessment, acoustic analysis, endoscopic imaging, and voice self-assessment.

The comparison between the CAPE-V and the self-assessment VAS scores at the pre- and post-treatment timepoints showed that the voice quality improved after ten CVM therapy sessions. This result is consistent with previous studies that showed that the CVM technique can improve the voice quality in patients with MTD [14, 15]. It is hypothesized that these results are due to musculoskeletal tension reduction and normalization of glottic and supraglottic contractions [11, 31].

In addition, the reduction of jitter and shimmer, as well as the increase of CPPS and MPT after the CVM therapy, reflects improved voice quality compared with before treatment. These findings agree with other studies [14, 15]. The contraction of circumlaryngeal and laryngeal muscles results in a limited range of motion of these muscles and consequently leads to acoustic changes in the patients with MTD. One of the critical areas in the larynx is the cricothyroid visor, which is controlled by the cricothyroid muscles and contributes to pitch changes during speech. This area is important for manipulation, and its contraction causes acoustic changes in speech [32]. In the CVM technique, phonation of high-pitch sounds, simultaneous with cricothyroid visor manipulation, can lead to an improvement in the acoustic features of the voice, which is due to the tension reduction of cricothyroid visor area leading to a decrease in voice pitch. In addition, reducing laryngeal activity and changing the voicing focus from laryngeal to forward focus improves voice quality [33].

Moreover, the videolaryngostroboscopy examination showed a decrease in the ML and AP contractions that were associated with in an increase in mucosal wave and the amplitude of vocal folds vibration after CVM treatment. This result indicated the effectiveness of this technique to improve the MTD symptoms. These results are compatible with the findings of previous studies [14, 15].

In this study, a potential association between COVID-19 and chronic dysphonia is suggested. The chronic dysphonia could be due to the persistent symptoms of pulmonary dysfunction. On the basis of earlier studies, the long-term respiratory complications of COVID-19 include chronic cough, fibrotic lung disease, bronchiectasis, and pulmonary vascular disease [17, 18]. Lechien *et al*. found a significant positive association between dysphonia and cough in patients with COVID-19 [5]. The effectiveness of CVM therapy on dysphonia associated with COVID-19 was investigated in the current study. It was hypothesized that this chronic dysphonia might greatly resist the therapy because of potential persistent respiratory complications. Nevertheless, the voice quality of the participant of this study got significantly better after ten CVM therapy sessions. It is added that the patient was advised to continue treatments until the dysphonia was completely resolved.

In this study, some limitations have to be considered. First, this study was carried out on one subject; thus, to further the understanding between the CVM therapy program and voice improvement of patients with MTD after COVID-19 symptoms, future studies with a larger sample size and control groups are required. Second, in this study, there was no follow-up assessment to investigate the maintenance of treatment outcomes. Third, as mentioned in the introduction section, voice therapy for MTD includes both direct and indirect aspects. In this study, we focused on the direct approach only. It is clear that, if two aspects are considered, the results may be better.

## Conclusions

In summary, the potential long-term health and communication consequences of COVID-19 are just beginning to be investigated. This case study has highlighted chronic dysphonia after COVID-19 infection and suggests that the CVM therapy approach may have positive outcomes for patients with MTD with this background.

## Data Availability

The datasets used and/or analyzed during the present study are available from the corresponding author on reasonable request.

## Abbreviations

WHO: World Health Organization
MTD: Muscle tension dysphonia
CVM: Cricothyroid visor maneuver
MPT: Maximum phonation time
VC: Vital capacity
PQ: Phonation quotient
